# Motion corrected fetal body magnetic resonance imaging provides reliable 3D lung volumes in normal and abnormal fetuses

**DOI:** 10.1002/pd.6129

**Published:** 2022-03-15

**Authors:** Joseph Davidson, Alena Uus, Alexia Egloff, Milou van Poppel, Jacqueline Matthew, Johannes Steinweg, Maria Deprez, Michael Aertsen, Jan Deprest, Mary Rutherford

**Affiliations:** ^1^ Department of Paediatric Surgery Evelina Children's Hospital London UK; ^2^ Elizabeth Garrett Anderson Institute of Women's Health University College London London UK; ^3^ GOS‐UCL Institute of Child Health London UK; ^4^ Biomedical Engineering Department School of Biomedical Engineering and Imaging Sciences King's College London London UK; ^5^ Centre for the Developing Brain School of Biomedical Engineering and Imaging Sciences King's College London London UK; ^6^ Department of Congenital Heart Disease Evelina Children's Hospital London UK; ^7^ Department of Imaging and Pathology Clinical Department of Radiology University Hospitals KU Leuven Leuven Belgium; ^8^ Clinical Department of Obstetrics and Gynaecology University Hospitals Leuven Leuven Belgium; ^9^ Academic Department of Development and Regeneration, Cluster Woman and Child KU Leuven Leuven Belgium

## Abstract

**Objectives:**

To calculate 3D‐segmented total lung volume (TLV) in fetuses with thoracic anomalies using deformable slice‐to‐volume registration (DSVR) with comparison to 2D‐manual segmentation. To establish a normogram of TLV calculated by DSVR in healthy control fetuses.

**Methods:**

A pilot study at a single regional fetal medicine referral centre included 16 magnetic resonance imaging (MRI) datasets of fetuses (22–32 weeks gestational age). Diagnosis was CDH (*n* = 6), CPAM (*n* = 2), and healthy controls (*n* = 8). Deformable slice‐to‐volume registration was used for reconstruction of 3D isotropic (0.85 mm) volumes of the fetal body followed by semi‐automated lung segmentation. 3D TLV were compared to traditional 2D‐based volumetry. Abnormal cases referenced to a normogram produced from 100 normal fetuses whose TLV was calculated by DSVR only.

**Results:**

Deformable slice‐to‐volume registration‐derived TLV values have high correlation with the 2D‐based measurements but with a consistently lower volume; bias −1.44 cm^3^ [95% limits: −2.6 to −0.3] with improved resolution to exclude hilar structures even in cases of motion corruption or very low lung volumes.

**Conclusions:**

Deformable slice‐to‐volume registration for fetal lung MRI aids analysis of motion corrupted scans and does not suffer from the interpolation error inherent to 2D‐segmentation. It increases information content of acquired data in terms of visualising organs in 3D space and quantification of volumes, which may improve counselling and surgical planning.

## Highlights

**What's already known about this topic?**

- Congenital diaphragmatic hernia (CDH) and congenital lung lesions (CLL) are prognosticated with ultrasound‐based measurements of the fetal lung in a single dimension; however true volumes may provide greater sensitivity for high risk cases. Current use of magnetic resonance imaging (MRI) to calculate fetal lung volumes is limited as two‐dimensional segmentation is labour intensive and risks interpolation and motion‐corruption errors.

What does this study add?

- Three‐dimensional lung volumes can be computed from deformable slice‐to‐volume registration (DSVR) 3D reconstructions and highly correlate with traditional 2D‐derived volumes. DSVR‐derived volumes, however, should be more reliable owing to higher resolution and semi‐automated calculations that do not rely on interpolation between slices on motion‐corrupted stacks.

## 1 INTRODUCTION

The antenatal work‐up of fetal thoracic anomalies increasingly includes fetal magnetic resonance imaging (MRI) in recent years. Fetal MRI aids in cases of diagnostic uncertainty by detailed delineation of soft tissues within the fetal chest, differentiating lung, bowel and liver more easily than ultrasound,^1^ helping in the assessment of congenital diaphragmatic hernia (CDH) and congenital lung lesions (CLL, including congenital pulmonary airway malformation [CPAM] and bronchopulmonary sequestration [BPS]). The currently standardised prognostic markers for CDH and CPAM are the observed/expected Lung Head Ratio [LHR] (O/E LHR^2^) and the CPAM volume ratio (CVR^3^), both measured by ultrasound. Both of these metrics utilise 2D measurements of aspects of the fetal chest to extrapolate volumes. Although these metrics are valid surrogates for total lung volume (TLV^4^), generation of such values may have inherent intra‐ and inter‐operator variability.^5^ Discrepancies between 2D ultrasound and MRI‐derived TLV measurements have been shown to be principally related to the failure to account for the contribution of the ipsilateral lung.^6^ Furthermore, ultrasound‐based imaging struggles with maternal habitus and fetal positioning, as well as extremes of liquor volume. 3D ultrasound lung volume measurements have been shown to be more difficult than by MRI, mainly because the most hypoplastic lung cannot be properly visualised.^7^ While US‐based measurements are undoubtedly the bedrock upon which assessment of fetal lung anomalies should be based, high volume fetal medicine centres have suggested that MRI‐calculated lung volumes may be a more accurate predictor of survival.^8^ This has not been formally proven and there is no standardised methodology for the use of MRI in these cases which limits the compilation of large datasets necessary for further study.^9^

Modern clinical fetal MRI protocols (primarily based on single shot turbo spin echo [ssTSE] sequence) allow fast acquisition of individual 2D slices that “freeze” fetal position in time. These slices have sufficiently high image and contrast resolution for diagnostic purposes in cases where fetal motion may previously have limited the information available. However, the misalignment between individual slices leads to corruption of volumetric information and loss of structural continuity within a slice stack. Therefore, output MRI stacks are termed “motion corrupted”. In general, to achieve a sufficient coverage of fetal visceral organs, clinical examinations require 3–6 MRI stacks acquired under different orientations with respect to the fetal body. The degree of motion corruption (misalignment of slices) can vary between the stacks and normally depends on the gestational age, amount of amniotic fluid, as well as fetal lie and mobility. It directly influences the accuracy of the fetal lung volume assessment, which is based on 2D slice‐wise segmentation of motion‐corrupted stacks followed by 3D interpolation. Therefore, calculated TLV values might vary for different stacks (from the same acquisition/the same subject) depending on the amount of motion corruption and acquisition plane. This is considered one of the limiting factors for MRI‐based TLV assessment. The common clinical practice for 2D MRI lung segmentation relies on selection of the least motion‐corrupted stack or averaging of the measurements obtained from multiple stacks.^9^ Furthermore, there are not existing standard guidelines regarding the degree of exclusion of vascular structures within the lungs or the consensus regarding the normogram formulas for O/E TLV assessment.

The recently proposed deformable slice‐to‐volume registration (DSVR) method^10^ is used for reconstruction of high‐resolution (e.g., 0.8 × 0.8 × 0.8 mm) isotropic 3D images of fetal body from multiple low‐resolution (e.g., 1.25 × 1.25 × 1.25 mm) motion‐corrupted stacks. In DSVR method, one of the low‐resolution stacks is selected as an initial target space and it is then registered to each of the slices using nonlinear free form deformation registration. This is followed by super‐resolution reconstruction (SR) of the 3D image from the registered slices. The full pipeline includes three interleaved SR and SVR steps. The resulting reconstructed images provide detailed 3D volumetric information and can be reoriented in any plane. This facilitates accurate 3D segmentations of fetal lungs and other organs allowing true volumetric analysis.

In this work, we sought to explore how 3D DSVR‐derived lung volumes would compare to those calculated by conventional manual 2D segmentation in cases of fetal thoracic anomalies, with a comparison to normal control cases.

## 2 METHODS

### 2.1 Case selection

The investigated cohort for 2D versus 3D TLV assessment included eight cases with abnormal lungs and eight control cases from the iFIND project [https://www.ifindproject.com] and clinical fetal cardiac MRI databases. The abnormal cases included six cases of CDH (one with concomitant BPS), and a further two cases of CLL (both macrocystic CPAM). The eight control cases were healthy control pregnancies and spanned approximately the same gestational range as the abnormal cases (22–32 weeks gestational age). In addition, 100 normal cases imaged as healthy control participants for research purposes were used for the general assessment of 3D DSVR‐derived fetal lung volumetry growth chart. These cases were selected by stratified random sampling in order to produce the widest possible range of gestational ages. Cases with extreme motion (i.e., >45° rotation in the body stacks) were excluded as their reconstruction is known to be challenging and may be prone to error, in practice this amounted to less than 5% of all cases in our database. All datasets used in this research were collected and processed subject to the informed consent of the participants.

### 2.2 Data

Each fetus was scanned using T2‐weighted ssTSE sequence, producing between 6 and 10 stacks with minimum of 5 orientations (orthogonal planes with respect to the uterus, fetal trunk and brain regions), with varying degrees of motion corruption (none severe as outlined above). No maternal breath hold or sedation was used during the acquisition. In all cases, images were acquired on an Ingenia 1.5 T (Philips) system using T2w ssTSE: Repetition time = 15,000 ms, Time to Echo = 80 ms, voxel size 1.25 × 1.25 mm, slice thickness 2.5 mm and spacing 1.25 mm. This acquisition protocol was optimised for both visualisation and 3D reconstruction at St. Thomas's Hospital and has been used for all clinical and research cases for more than 5 years. The consent under which they were performed, mean that linkage to clinical data (i.e., FMU‐performed US with O/E LHR) was not possible.

For the initial 16 study cases, 2D slice‐wise segmentation of the fetal lungs was performed in ITK‐SNAP (http://www.itksnap.org) in the axial stack with respect to the fetal trunk and a total fetal lung volume was calculated using the conventional interpolation approach. On average, the manual 2D‐slice wise lung segmentation took 5–10 min per case depending on the on gestational age (GA) of the subject and presence of anomalies. The segmentations were performed by a single researcher trained in fetal MR image processing.

These datasets also underwent DSVR reconstruction to 0.85 × 0.85 × 0.85 mm resolution using SVRTK toolbox (https://github.com/SVRTK/SVRTK) and lung volumes were calculated after automated 3D segmentation followed by manual refinement in 3D Slicer (https://www.slicer.org). The automated segmentation was based on a 3D U‐Net convolutional neural network for lung segmentation,^11^ pretrained on 60 manual segmentations of DSVR‐reconstructed images. The network is operational on both normal and abnormal cases. The total time for the automated segmentation with additional manual refinement took less than 5 min per case with the additional editing required primarily for the abnormal cases (hypoplastic lung or lesions).

**FIGURE 1:**
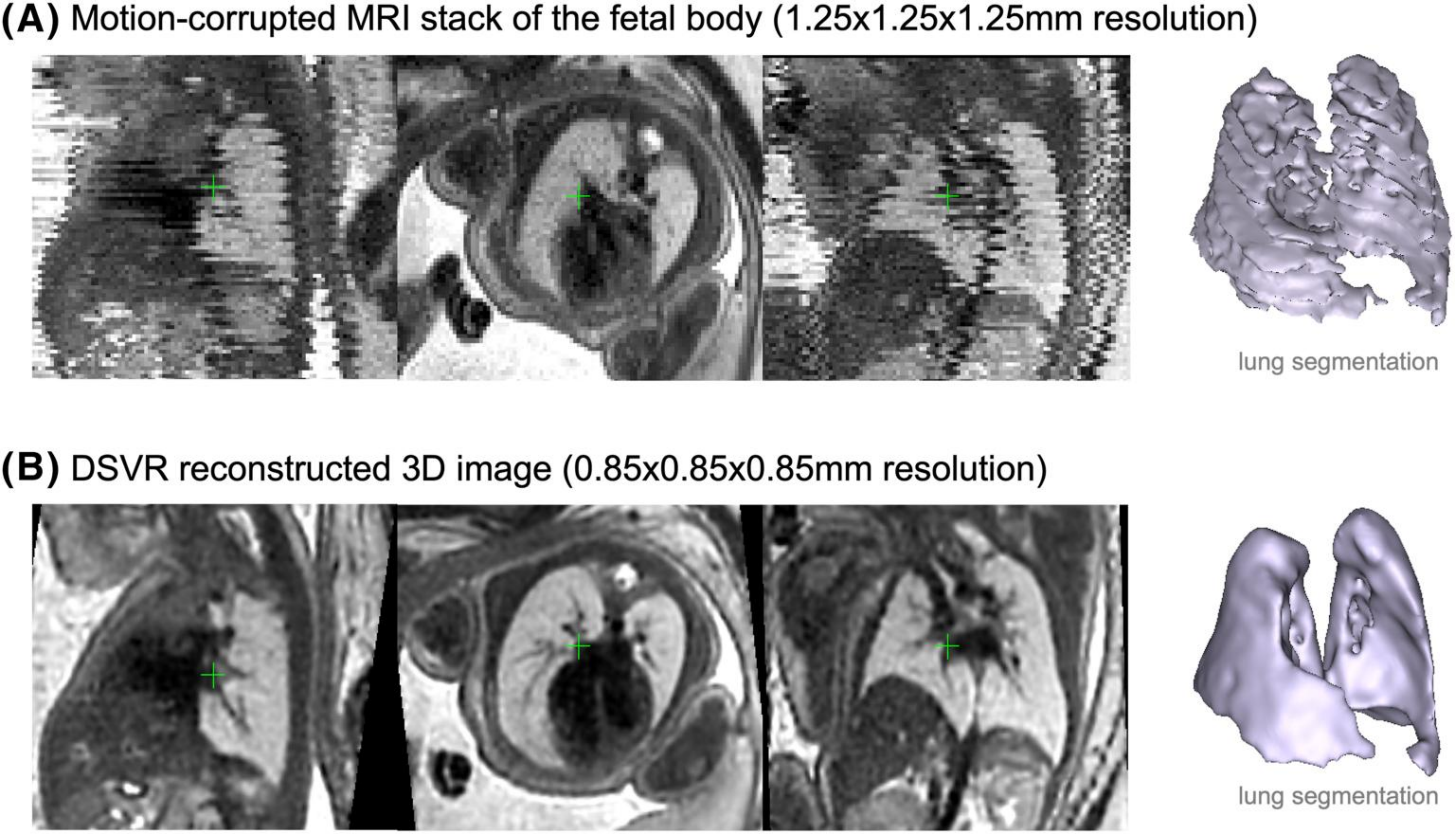
Example of a motion corrupted stack and the corresponding 3D deformable slice‐to‐volume registration (DSVR) reconstructed image along with the output lung segmentations

An example of one of the original 2D axial stacks affected by minor to moderate motion and the corresponding 3D DSVR reconstruction are displayed side by side for descriptive purposes in Figure 1 and Supplementary Video 1. The processing of the additional 100 cases included reconstruction using the standard DSVR pipeline^10^ followed by semi‐automated lung segmentation with additional manual refinement. Our experience is that DSVR provides good reconstruction quality in >95% of datasets (including those referred from external centres) and that reconstruction time takes 10–45 min^10^ on average depending on the GA of the subjects and number of stacks. Lung volumes from the 16 cases derived from 3D DSVR and the conventional 2D‐based approach underwent pairwise Bland–Altmann comparison to assess for bias in measurement. In cases of CLL, lesion volume was calculated and is provided here in cm^3^ and as a proportion of TLV. A normal curve (growth chart) for fetal lung volumes was generated for 21–32 weeks GA range from 100 DSVR reconstructions similarly as has been reported by Meyers et al. based upon their own 2D‐segmentation data.^12^ The resultant curve was depicted graphically along with previously published MRI‐generated fetal lung volume normograms.^12^, ^13^ As this was a pilot study, there was no power calculation performed and no further statistical analysis was deemed appropriate to perform.

## 3 RESULTS

**FIGURE 2:**
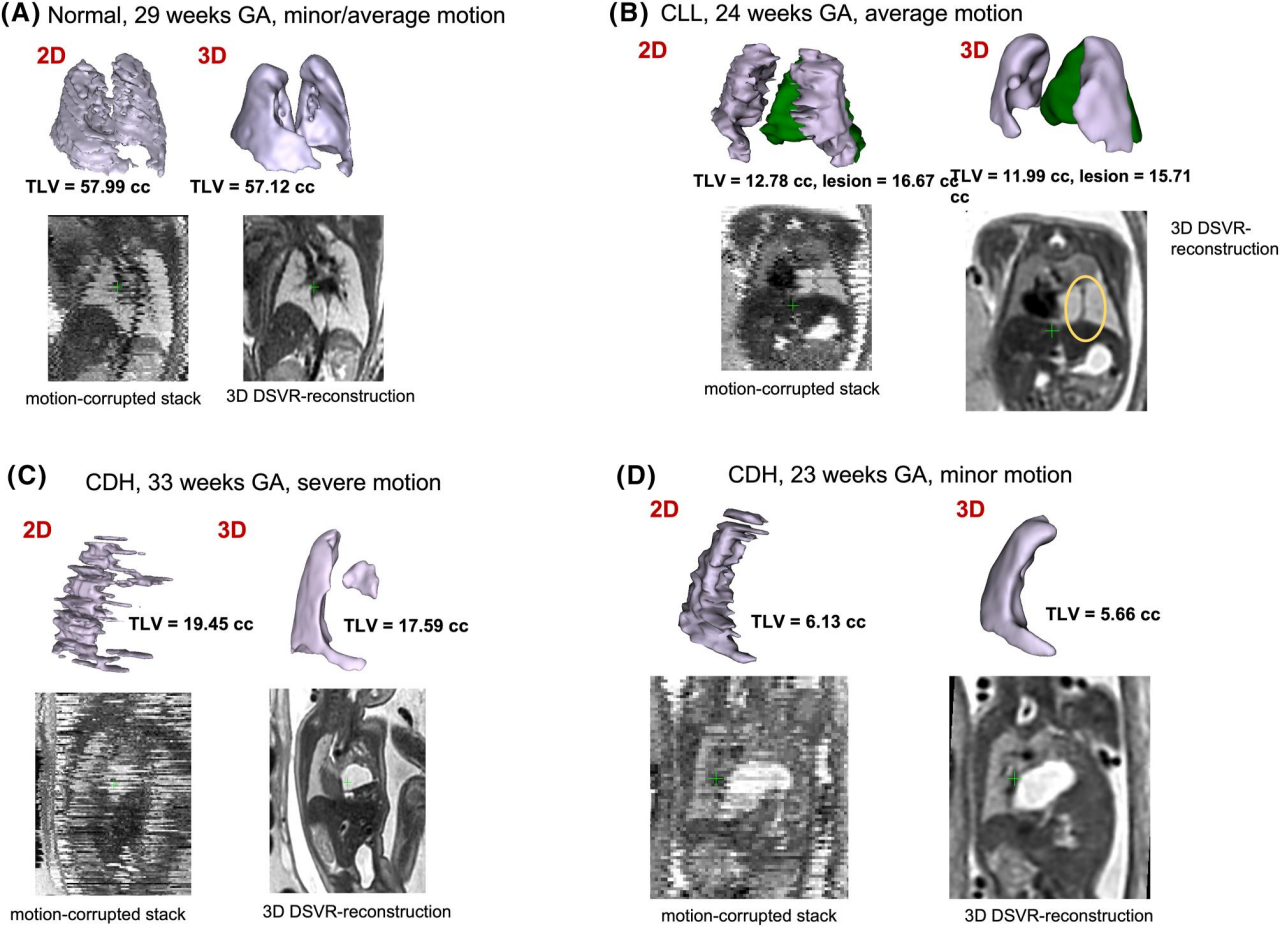
Example cases of 2D‐manual and automated deformable slice‐to‐volume registration (DSVR) 3D segmentation of the fetal lung (lilac in all cases). (A) normal fetus at 29 weeks gestation with mild to moderate motion corruption. (B) 24 weeks gestation fetus with a left lower lobe lesion – segmentation of lesion in green, a systemic feeding vessel can be visualised on the DSVR images, suggesting a bronchopulmonary sequestration (BPS) or hybrid‐type lesion. (C) 33 weeks gestation fetus with left congenital diaphragmatic hernia (CDH), severe motion corruption of the input stack is resolved with DSVR. (D) 23 weeks gestation fetus with left CDH with severe hypoplasia of the left lung as defined by DSVR reconstructed segmentation

The examples of 2D versus 3D lung segmentations for 4 cases are depicted in Figure 2; motion corruption of the axial‐plane stack can clearly be appreciated in the coronal plane, with the coronal section of the DSVR shown alongside each case. In should be noted that the employed thin slice acquisition protocol with 1.25 slice spacing provides denser sampling of the lungs thus ensuring higher spatial coverage. However, it is also more susceptible to motion artifacts in comparison to the conventionally used 3 mm spacing due to the longer scanning times.

**FIGURE 3:**
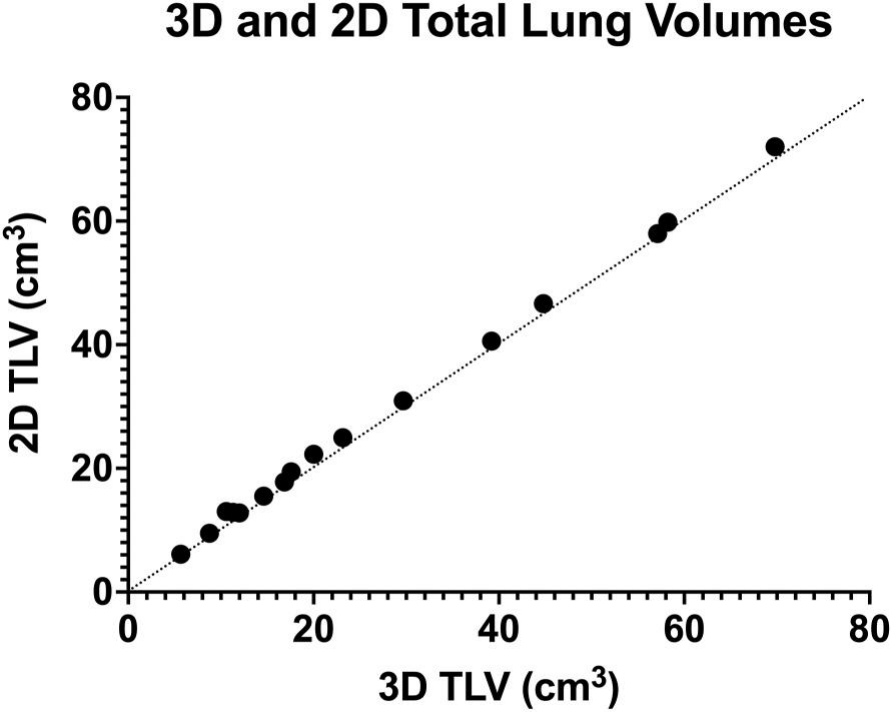
2D manual segmentation derived total lung volume (TLV) plotted against deformable slice‐to‐volume registration (DSVR)‐derived 3D TLV. Bland–Altman bias −1.44 cm^3^. For reference the line *y* = x is also drawn (i.e., perfect match of 2D and 3D volumes)

In comparison, DSVR was able to produce clear 3D images even from severely motion‐corrupted stacks (Figure 2C) and the resultant images can be examined in any plane and at a higher resolution than that of the original input stack (Figure 1, Video 1). The resultant segmentation of fetal lungs demonstrates a smooth outline, with normal cases depicting sufficient detail even to appreciate the lingula (Figure 2A) or a systemic feeding vessel in cases of CLL (Figure 2B). Comparison of the volumes generated from 2D and 3D segmentations is given in Figure 3; results correlated strongly, however a consistently lower measurement was made with 3D segmentation (Bland–Altman: Bias −1.44 cm^3^ [95% confidence limits −2.63 to −0.24]). This was felt to be due to the enhanced ability to exclude structures of the pulmonary hilum in 3D reconstructions, as well as an inherent reduction in interpolation error by accommodating motion artefact.

**FIGURE 4:**
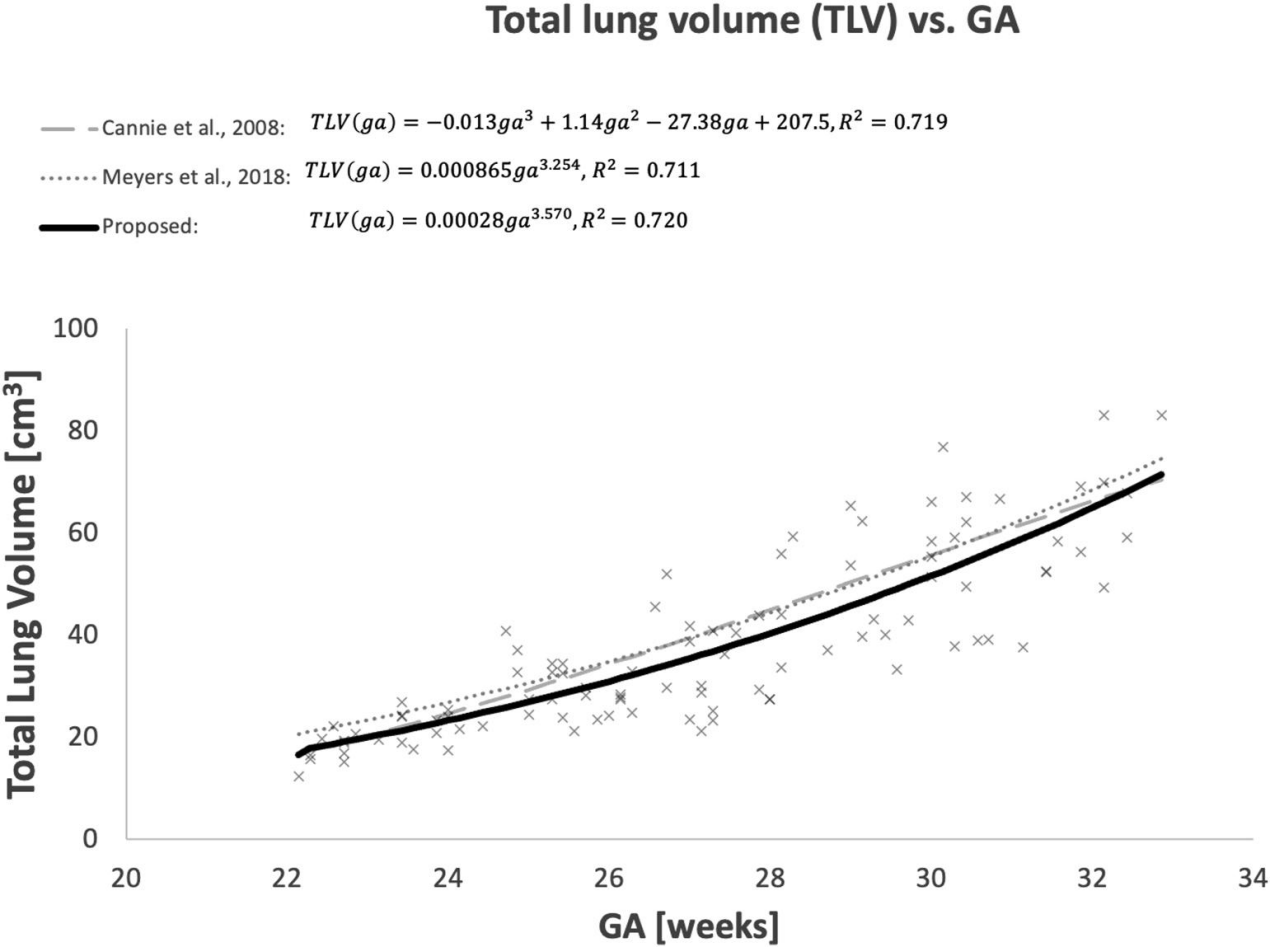
Normal total lung volumes as calculated by DSVR; 100 normal cases were used to generate a curve with the equation TLV(ga) = 0.00028ga^3.57^, with an R^2^ = 0.72. For reference the curves suggested by Cannie^13^ and Meyers^12^ are included on the same graph

Having defined the fidelity of DSVR‐derived 3D TLV, we proceeded to produce a normal curve based upon 100 healthy cases undergoing fetal MRI as normal controls for research, presented in Figure 4. The corresponding fitted TLV normogram was similar to the previously reported gestational TLV curves produced from conventional 2D‐based segmentation data by Cannie et al^13^ and Meyers et al.^12^ The generated regression line had a similar *R* ^2^ (0.72) to those published and was subsequently used to generate the “Expected” value for the depiction of Observed:Expected TLV in the abnormal cases.

**FIGURE 5:**
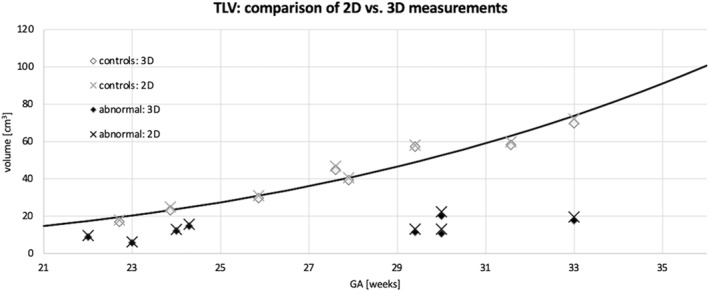
Total lung volumes of normal and abnormal cases computed from 3D deformable slice‐to‐volume registration (DSVR)‐derived and 2D manual slice‐wise segmentations with reference to our DSVR‐derived normogram

The manually segmented 2D lung volumes and corresponding 3D DSVR‐derived lung volumes are depicted graphically in Figure 5 with reference to the DSVR‐derived normal curve. Table 1 displays data calculated from anomaly cases. CPAM lesion volumes were calculated from DSVR reconstructions and are presented as a percentage of the remaining TLV; of note one case had CDH and a BPS of the lung on the same side as the hernia, this case had significantly smaller lesion volume related to lung volume.

**TABLE 1:** Abnormal cases included in the study

Case	Uss assessment	2D‐Observed TLV (CM^3^)	3D‐Observed TLV (CM^3^)	2D O/E TLV	3D O/E TLV
CDH 1	Left sided, liver up	19.45	17.59	25.7%	23.8%
CDH 2	Left sided, liver up	6.13	5.66	26.3%	27.8%
CDH 3	Left sided, liver up	22.28	20.01	40.2%	38.1%
CDH 4	Left sided, liver up	12.90	11.30	24.9%	23.1%
CDH 5	Right sided, liver down	13.01	10.57	23.3%	20.1%
CDH + BPS (lesion vol. 2.83 cm^3^ [32%TLV])	Right sided, liver down left‐sided BPS	9.50	8.76	47.0%	50.4%
CPAM1 (lesion vol. 16.86 cm^3^ [140%TLV])	Macrocystic unifocal CPAM	12.78	11.99	47.7%	50.6%
CPAM2 (lesion vol. 21.59 cm^3^ [148%TLV])	Macrocystic unifocal CPAM	15.52	14.61	55.6%	59.1%

*Note*: Ultrasound based assessment with calculated observed/expected lung:head ratio. 2D (manual 2D segmentation) and 3D (DSVR‐derived) O/E TLV values are computed based on the Meyers 2D‐derived and the currently proposed 3D DSVR‐based normograms; lesion volumes for CLL are also presented as a percentage of the 3D TLV.

Abbreviations: BPS, Broncopulmonary Sequestration; CDH, Congenital Diaphragmatic Hernia; CPAM, Cystic Pulmonary Airway Malformation; TLV, Total Lung Volume.

## 4 DISCUSSION

This pilot study demonstrates that MRI of the fetal thorax can be processed by 3D DSVR reconstruction, and subsequent lung volumetry can be performed with volumes generated that highly correlate to the “gold standard” 2D‐manual segmentation derived volumes currently used in clinical practice for O/E TLV assessment. Furthermore, 3D lung segmentations potentially provide more accurate TLV estimation since they minimise the errors on the conventional 2D‐based approach: segmentation of 2D stacks and subsequent volumetric measurements inherently cannot be regarded as “true” to the subject, since a degree of motion corruption is always present in these acquired images and the slice‐to‐slice measurements will require interpolation in order to produce workable volumes. The Bland–Altman analysis of the quantitative results as well as the general visual inspection of 2D versus 3D segmentations suggest that using 3D DSVR‐based assessment may minimise these errors as the resultant images are not only of a higher resolution, but also aprovide continuous 3D information does not affected by interpolation. This means that small structures of the thorax, such as hilar vessels, can be reliably excluded in all cases, producing volume measurements far closer to that of the patient. This potentially resolves one of the current limitations to the validity of MRI derived volumes in predicting outcomes as reliably as LHR.^14^

Three dimensional images produced from MRI of the fetus have been conclusively demonstrated to enhance diagnostic capabilities of MRI in assessment of the fetal brain.^15^ This pilot series demonstrates that DSVR method provides the means for accurate calculation of lung volumes for both normal and abnormal cases. This is especially useful for CDH cases for more accurate evaluation of the observed to expected lung volume ratios and the volume of hypoplastic lung. The recent advances in image processing methods reduce variability in segmentation,^16^ further improving the reliability of the volumetry results. We anticipate that MR based lung volumetry may soon be validated as a reliable and more accurate prognostic indicator than the current use of the LHR; since it would invariably more closely predict true TLV. Furthermore, the ability to outline lesion volumes in cases of CLL will likely produce a prognostic marker more faithful to true lesion size than the currently utilised CVR where cross sectional areas are utilised to estimate lesion volume relative to fetal size. The consistency of such a method will certainly deliver on the recent plea for consistency in prognostication of these cases.^9^ It should be noted that although we have provided lesion volume proportionate to TLV, it would be possible to segment any aspect of the fetus and derive a corresponding volume ratio proportional to this as well as the relative position of the organs (e.g., the degree of liver herniation or position of the stomach in CDH cases). Furthermore, automation of these 3D measurements using deep learning tools will potentially allow unbiased analysis for large cohorts.

Recently, it has also been demonstrated that DSVR reconstructed MRI images may provide additional diagnostic information for complex fetal anomalies.^1^ The illustrative cases included within this dataset also show that DSVR will produce datasets that are more accessible to the clinician by resolving motion corruption with a resolution superior to that of the original input stacks. This will certainly aid in prenatal counselling and planning of the operative approach in neonatal thoracic surgery; the reliable depiction of systemic feeding vessels in BPS, combined with US Doppler assessment may reduce the reliance on early postnatal CT imaging.^17^

Regarding longitudinal assessment of CDH or CLL, it should be recognised that the repeated use of MRI is considerably more expensive, time consuming and lengthy compared to an expert‐performed ultrasound. Therefore, we anticipate that there will continue to be a role for ultrasound in the monitoring of such conditions to delivery. Indeed, serial CVR assessment has recently been shown to be effective in predicting a need for perinatal intervention in cases of CLL.^18^

## 5 CONCLUSIONS

This pilot study demonstrates the potential of a 3D reconstruction platform such as DSVR in the analysis of the fetal lung, with an emphasis on accuracy and reliability in image derived volumetry. We anticipate that MR based lung volumetry may soon be validated as a reliable and more accurate prognostic indicator than the current use of the LHR.

Our current work focuses on deep learning methods in order to provide reliable volumetric assessment for a wide range of anomalies as well as optimisation of different MRI acquisition protocols. This will allow integration of both DSVR and volumetry pipelines in different clinical centres that employ fetal MRI on a regular basis.

## CONFLICT OF INTEREST

None of the authors have any conflict of interest to declare.

## ETHICS STATEMENT

This study received national ethics approvals through the NHS REC: [07/H0707/105] in 2007 and [14/LO/1806] in 2014. All participating pregnant women taking part consented for the use of their imaging data in research.

## Supporting information

Supplementary Information 1

## Data Availability

The code for DSVR is available to all at https://github.com/SVRTK/SVRTK. Any reader wishing to explore its use is welcome to contact Alena Uus (alena.uus@kcl.ac.uk).
